# Cost-Effectiveness of Facilitated Access to a Self-Management Website, Compared to Usual Care, for Patients With Type 2 Diabetes (HeLP-Diabetes): Randomized Controlled Trial

**DOI:** 10.2196/jmir.9256

**Published:** 2018-06-08

**Authors:** Jinshuo Li, Steve Parrott, Michael Sweeting, Andrew Farmer, Jamie Ross, Charlotte Dack, Kingshuk Pal, Lucy Yardley, Maria Barnard, Mohammed Hudda, Ghadah Alkhaldi, Elizabeth Murray

**Affiliations:** ^1^ Mental Health and Addiction Research Group Department of Health sciences University of York York United Kingdom; ^2^ Cardiovascular Epidemiology Unit Department of Public Health and Primary Care University of Cambridge Cambridge United Kingdom; ^3^ Nuffield Department of Primary Care Health Sciences University of Oxford Oxford United Kingdom; ^4^ Research Department of Primary Care and Population Health University College London London United Kingdom; ^5^ Department of Psychology University of Bath Bath United Kingdom; ^6^ Department of Psychology University of Southampton Southampton United Kingdom; ^7^ Department of Diabetes & Endocrinology Whittington Health NHS Trust London United Kingdom; ^8^ Population Health Research Institute St. George’s University of London London United Kingdom; ^9^ Community Health Sciences Department College of Applied Medical Sciences King Saud University Riyadh Saudi Arabia

**Keywords:** cost-effectiveness, type 2 diabetes mellitus, self-management, internet

## Abstract

**Background:**

Type 2 diabetes mellitus is one of the most common long-term conditions, and costs health services approximately 10% of their total budget. Active self-management by patients improves outcomes and reduces health service costs. While the existing evidence suggested that uptake of self-management education was low, the development of internet-based technology might improve the situation.

**Objective:**

To establish the cost-effectiveness of a Web-based self-management program for people with type 2 diabetes (HeLP-Diabetes) compared to usual care.

**Methods:**

An incremental cost-effectiveness analysis was conducted, from a National Health Service and personal and social services perspective, based on data collected from a multi-center, two-arm individually randomized controlled trial over 12 months. Adults aged 18 or over with a diagnosis of type 2 diabetes and registered with the 21 participating general practices (primary care) in England, UK, were approached. People who were unable to provide informed consent or to use the intervention, terminally ill, or currently participating in a trial of an alternative self-management intervention, were excluded. The participants were then randomized to either usual care plus HeLP-Diabetes, an interactive, theoretically-informed Web-based self-management program, or to usual care plus access to a comparator website containing basic information only. The participants’ intervention costs and wider health care resource use were collected as well as two health-related quality of life measures: the Problem Areas in Diabetes (PAID) Scale and EQ-5D-3L. EQ-5D-3L was then used to calculate quality-adjusted life years (QALYs). The primary analysis was based on intention-to-treat, using multiple imputation to handle the missing data.

**Results:**

In total, 374 participants were randomized, with 185 in the intervention group and 189 in the control group. The primary analysis showed incremental cost-effectiveness ratios of £58 (95% CI –411 to 587) per unit improvement on PAID scale and £5550 (95% CI –21,077 to 52,356) per QALY gained by HeLP-Diabetes, compared to the control. The complete case analysis showed less cost-effectiveness and higher uncertainty with incremental cost-effectiveness ratios of £116 (95% CI –1299 to 1690) per unit improvement on PAID scale and £18,500 (95% CI –203,949 to 190,267) per QALY. The cost-effectiveness acceptability curve showed an 87% probability of cost-effectiveness at £20,000 per QALY willingness-to-pay threshold. The one-way sensitivity analyses estimated 363 users would be needed to use the intervention for it to become less costly than usual care.

**Conclusions:**

Facilitated access to HeLP-Diabetes is cost-effective, compared to usual care, under the recommended threshold of £20,000 to £30,000 per QALY by National Institute of Health and Care Excellence.

**Trial Registration:**

International Standard Randomized Controlled Trial Number (ISRCTN) 02123133; http://www.controlled-trials.com/ISRCTN02123133 (Archived by WebCite at http://www.webcitation.org/6zqjhmn00)

## Introduction

### Background

There is a global epidemic of diabetes mellitus, with an estimated 10% of the global population, or 422 million people, affected. Around 90% of these people have type 2 diabetes mellitus (T2DM) [1]. The personal and health care costs of T2DM are substantial. Recent estimates suggest that about 11% of the total global health expenditure is due to diabetes [2]. Most of these costs are due to preventable complications [3]. Also, diabetes also results in societal costs, including the cost of missed workdays [4], personal costs, including out of pocket costs [2], and the impact on employment and earnings potential [5,6]. Patient education and self-management support has been identified as a priority for global health in recent years [7] and has the potential to both improve outcomes and reduce costs [8]. However, internationally, uptake of self-management education remains low [9], partly due to logistical problems with attending courses [10].

Web-based self-management support has the potential to increase uptake by overcoming some of the logistical problems associated with other forms of delivery as it can be accessed at home, at the user’s convenience. We have developed a comprehensive, evidence-based, theoretically informed, Web-based self-management program for adults with T2DM called Healthy Living for People with Type 2 Diabetes (HeLP-Diabetes). Overall content was guided by the Corbin and Strauss model, which hypothesizes that patients must undertake medical, emotional and role management in dealing with a long-term condition [11]. If effective, Web-based interventions have the potential to be highly cost-effective, as they can be delivered at scale across large populations, with relatively low additional costs per additional user [12], unlike telephone-based or face-to-face education where labor costs account for a substantial proportion of total cost [13].

We undertook an individually randomized controlled trial in primary care to determine the effectiveness and cost-effectiveness of HeLP-Diabetes compared to a simple, text-based website, all with the access to usual care for people with T2DM [14]. The aim of this paper is to present the health economic analysis of this comparison based on the data collected in the trial and to examine the cost-effectiveness of facilitated access to HeLP-Diabetes. The analysis was undertaken from the collective perspective of the National Health Services (NHS) and personal social services (PSS), following the National Institute of Health and Care Excellence (NICE) guidance [15], as the two share the same resources. The results on clinical effectiveness are reported in a separate article previously published [16]. All costs are presented in pounds sterling (£) 2014 prices.

## Methods

### Approval and Ethical Considerations

Ethics approval was obtained from Camden and Islington National Research Ethics Service (NRES) committee, reference 12/LO/1571.

### Design, Setting, and Participants

The HeLP-Diabetes trial was a multi-center, two-arm individually randomized controlled trial carried out in primary care settings in England, United Kingdom (UK). The detailed trial design was fully reported in the published protocol [14], and the clinical effectiveness article [16]. There were no changes to the methods after the protocol was agreed and the start of the trial. Twenty-one general practices from across England participated, with a mix of urban, suburban, and rural practices. Adults, aged 18 years or over, registered with participating practices, and diagnosed with T2DM were eligible for inclusion in the trial. People who were unable to provide informed consent (eg, due to psychosis or cognitive impairment), unable to use the intervention (eg, due to physical, sensory or intellectual impairment, or inability to understand basic spoken or written English), terminally ill, or currently participating in a trial of an alternative self-management intervention, were excluded. There were no exclusions based on the duration of diagnosis, level of diabetes control, previous experience of self-management education, computer and internet experience, or access to the internet at home.

Eligible participants were briefed on the trial by a practice or research nurse (See the Patient Information Sheet in Multimedia Appendix 1) and then individually randomized to either the intervention or control group using Web-based randomization independently of the trial team. Randomization was conducted in a 1:1 ratio using random permuted blocks of sizes 2, 4, and 6, stratified by recruitment center. Participants were informed the trial compared two forms of Web-based support but were blinded as which was the intervention and which the comparator. Each participant had access to their allocated intervention for 12 months after randomization. Follow-up was undertaken at 3 and 12 months postrandomization.

#### Intervention

The intervention consisted of facilitated access to HeLP-Diabetes. Facilitation consisted of an introductory training session with practice nurses. In this session, patients were shown on a computer how to log on and set a username and password, and introduced to the structure, contents, and features of the website and how to navigate it. A booklet summarizing the information introduced in the session was given to the patients to take home.

HeLP-Diabetes was a theoretically informed, Web-based program, whose overall goals were to improve health outcomes and reduce diabetes-related distress [17]. It was developed using participatory design principles, with substantial input from patients with T2DM and health professionals caring for such patients. The content was designed to be accessible to people with a wide range of literacy and health literacy skills, with all essential content provided in both video and text. The content sections covered information on diabetes as a medical condition and its impact on people’s life; behavior change components to support adoption of healthier lifestyles; and a third strand of components focusing on emotional well-being based on cognitive behavioral therapy and mindfulness.

The program also included an online forum where the participants could post and share their questions, concerns, and experiences. There was also an “Ask the Expert” facility, where questions were reviewed and responded to by a multi-disciplinary team including an information scientist, clinicians, and patient representatives (see Multimedia Appendix 2). The forum was monitored daily by both research staff and patient representatives.

Engagement with the program was promoted through regular newsletters, emails and mobile text messages containing updates on latest diabetes-related research or practice, seasonally-relevant advice, and links to specific relevant parts of the program. A medical information scientist reviewed the diabetes-related research published each month and provided a summary of the important, useful or relevant research. The summary was then discussed by a team of clinicians, psychologists, health service researchers and patient representatives before selected items of interest were written up for a patient audience. A more detailed description of the intervention is provided in a separate clinical article [16] and a National Institute for Health Research (NIHR) monograph [18].

#### Comparator

HeLP-Diabetes was designed to be provided as an addition to current practice. However, to improve acceptability to participants and to maintain blinding, all participants were assigned access to a website. Participants in the control group were given access to a simple information website, based on the information readily available in the public domain on the website of the main UK diabetes charity (Diabetes UK) or National Health Service patient information website (NHS Choices). Participants in the control group were also given an introductory facilitation meeting, in which they were shown how to navigate the website, and an information booklet to take home.

#### Health Outcomes

The health outcomes for the health economic analysis were diabetes-related distress, measured by the Problem Areas in Diabetes (PAID) questionnaire, and quality of life, measured by EQ-5D-3L. The PAID questionnaire consisted of 20 items focusing on areas that cause difficulty for people living with diabetes, including social situations, food, friends and family, diabetes treatment, relationships with health care professionals and social support [19]. PAID scores range from 0-100, with lower scores indicating less stress, with a score of 40 or more indicating significant distress. EQ-5D-3L is a standardized instrument for measuring health-related quality of life, which has five domains (5D), each with three levels (3L) measuring daily difficulties in that domain [20]. Both these self-reported outcome measures were collected online at baseline, three months, and 12 months follow-up. The tariff for each combination of the EQ-5D-3L levels for the UK population was applied to calculate utility values [21]. The utility values range from –0.594 to 1, with higher values indicating better quality of life. Quality-adjusted life years (QALYs) were then calculated over the duration of the trial using the area under the curve of utility values from the three time points [22]. QALYs were not discounted because the assessing period was 12 months.

#### Costs of the Intervention

There were two types of costs related to the intervention: those incurred during the development and optimization of the intervention; and those related to ongoing delivery and maintenance of the intervention. Development costs were not taken into account for this analysis. As per NICE guidance [15], the evidence on costs should relate to the National Health Services (NHS) and personal social services (PSS) resources. The development costs, in this case, although considerable, related to research funding rather than NHS and PSS resources, and unlikely to be repeated if the intervention is adopted in practice.

The costs relating to ongoing maintenance and delivery of the intervention within the trial form the basis for the current analysis. These consisted of: the cost of delivery of the intervention; the cost of maintenance and updating of the intervention; and cost of facilitating activities undertaken to improve uptake and use. If the intervention were to be widely implemented into routine health care, all these activities would be required on an on-going basis. Therefore, the costs of these activities that occurred during the trial were used to estimate the real costs in practice.

Delivering, maintaining, and updating the intervention involved two types of costs: costs related to hardware and software; and staff costs. Staff costs related to activities for engagement, moderating the online forum, revising the content of the website, and responding to ‘Ask the Expert’ questions. Staff costs were also incurred by the third-party service provider responsible for hosting and maintaining the intervention.

Costs related to hardware, software, and work undertaken by the third-party service provider were recorded from actual invoices. These included a weekly review of recent development in the field, domain names purchase, website hosting, quarterly maintenance of the website, and Security Socket Layer certificate purchase. Some of the third-party services were contracted for longer than 12 months, so costs were calculated for a one-year period based on the invoiced amount and their length of service.

Activities not undertaken by the third-party providers were carried out by either professional staff or by patient representatives. Costs related to work undertaken by patient representatives were recorded from the payments made to representatives, who were reimbursed for their time in line with INVOLVE guidance [23]. INVOLVE is a NIHR funded national advisory group to support active public involvement in NHS, public health and social care research. These included patient representatives’ feedback on development review, forum monitoring, and their review of feedback from the clinical team. Costs related to activities undertaken by research staff and clinicians were estimated from workloads during the trial period, by recording the time taken for each activity, the frequency of that activity and the number and grade of staff involved. These included writing and sending emails, short message service (SMS), and newsletters, forum monitoring, interaction with patients on the website, and contents review and update. The costs were then calculated by multiplying the time spent by the average wage for each type of staff member. Hourly costs for research staff were taken from the academic pay scale [24], and hourly costs for General Practitioners (GPs) were taken from unit costs of health and social care edited by Personal Social Services Research Unit (PSSRU) [25].

Participants in both groups were provided with facilitation by practice or research nurses to encourage use and uptake of the intervention and comparator. Participants were also provided with a booklet, summarizing the training they had received (login details, how to use the website). All participants were provided with this introductory session and booklet. As this activity was not required for the comparator website and was only undertaken to maximize comparability between the intervention and the comparator, we considered it a research activity and assumed that no intervention costs were incurred in the control group.

Practice/research nurses required training in providing this introductory session. Each nurse attended an hour face-to-face training session provided by a member of the research team. They were also provided with printed training materials, reminding them how to register patients and how to introduce the website. The costs of the training and introductory session were calculated from the time spent on each activity by the nurses and research team staff, plus travel time for the research team, multiplied by their respective hourly salary rates. In this case, we took the hourly rate of practice nurses for all nurses’ time. The costs of the printed training materials and the booklets issued to participants were obtained from invoices from the printers.

All intervention costs were allocated to the participants in the intervention group of the trial to give a per participant cost.

#### Health Service Resource Use

Health care resource use, including primary, secondary and community services, was collected for both groups using bespoke service use questionnaires. The majority of information about service use and participants’ prescriptions were extracted from participants’ medical records by practice or research nurses. The remainder of the service use data were collected retrospectively from participants using a self-report questionnaire online. These data were collected at baseline for the 12 months period before the trial, at three months follow-up for the three months period after randomization and at 12 months follow-up for the nine months after three months follow-up. The quantities were then multiplied by a set of national average unit costs (Table 1). The total costs of health services were then summarised at an aggregated level, (ie, costs of health services use from data provided by nurses, medication costs from data provided by nurses, and costs of health and social services use from data provided by participants), for the corresponding periods respectively. Any missing data on individual services resulted in a missing cost for the entire section.

Where applicable, Value Added Tax at 20%, salary on-costs, and overheads were added. Unit costs for out-of-hours services were estimated based on a national audit [26], assuming the duration of consultations was the same as for in-hours services. Data on travel time for home visits were not available, so we adopted the assumed 12 minutes per visit estimate made by PSSRU 2014 [25]. No allowance was made for travel expenses.

Due to the large amount of medications taken by this particular population, only current prescriptions taken at the time of data collection were extracted. Prescribed items were matched with the Prescription Cost Analysis England 2014 [28] for a cost per item, using their generic name, form, and strength where available. In the absence of full information, a weighted average cost per item was calculated based on available information. Unless it was specified that no medication was prescribed, blank entries were considered as missing data. We also assumed that all prescriptions were for chronic conditions and issued for one month at a time over the corresponding period. Costs were not discounted as the assessing period was 12 months.

**Table 1:** National average unit cost used in the analysis.

Health Service Use		Unit cost (£) (per consultation or per episode)	Sources
**GP^a^ consultation**			
	In surgery	38	PSSRU^b^ 2014 [25]
	Home visit	62	PSSRU 2014 [25]
	Telephone	23	PSSRU 2014 [25]
**Practice nurse consultation**			
	In surgery	11	PSSRU 2014 [25)
	Home visit	18	PSSRU 2014 [25]
NHS^c^ Walk-In Clinic		56	PSSRU 2014 [25]
**Out-of-hour services**			
	Telephone advice	36	Out-of-hours GP services in England [26], PSSRU 2014 [25]
	Home visit	117	Out-of-hours GP services in England [26], PSSRU 2014 [25]
	In surgery	86	Out-of-hours GP services in England [26], PSSRU 2014 [25]
Accident and emergency service admission		167	Reference Costs 2013-14 [27]
Podiatrist		44	Reference Costs 2013-14 [27]
Optometry		97	Reference Costs 2013-14 [27]
Physiotherapy		46	Reference Costs 2013-14 [27]
**Counselling**			
	Primary care	46	PSSRU 2014 [25]
	Community	138	Reference Costs 2013-14 [27]
Clinical test		2	Reference Costs 2013-14 [27]
Outpatient appointment		111	Reference Costs 2013-14 [27]
Day case		698	Reference Costs 2013-14 [27]
Inpatient admission		1891	Reference Costs 2013-14 [27]
**District nurse**			
	Home visit	46	Reference Costs 2013-14 [27], PSSRU 2014 [25]
	In surgery or clinic	37	Reference Costs 2013-14 [27]
Social worker		55	PSSRU 2014 [25]
Occupational therapy		64	Reference Costs 2013-14 [27]
Dietician		80	Reference Costs 2013-14 [27]

^a^GP: general practitioner.

^b^PSSRU: Personal Social Services Research Unit.

^c^NHS: National Health Service.

### Analysis

#### Missing Data

Multiple imputation was used as the primary method to account for missing data at both baseline and follow-up. A chained equation model was developed, and predictive mean matching was used as the imputation method for continuous variables, using the five nearest neighbors to the prediction as a set to draw from. All missing data were imputed separately by trial group. The imputation was performed at the aggregated level (ie, costs of health services use from data provided by nurses, prescription costs from data provided by nurses, costs of health and social services use from data provided by participants, PAID score and EQ-5D-3L utility value from data provided by participants). PAID and EQ-5D-3L data collected outside of the pre-specified “window” of 10-14 months following randomization were considered invalid as 12-month outcomes and only used as imputing factors along with baseline and other outcome data. The percentage of missing data served as the base of the number of imputations, as a rule of thumb [29].

#### Primary Analysis

The primary analysis followed a pre-specified analysis plan, comparing the groups as randomized (intention-to-treat). It focused on a within-trial analysis of costs and benefits, with no projected time horizon. A linear mixed effects model was fitted with the 12-month outcome as the dependent variable, adjusting for the baseline variables age, sex, presence of pre-existing cardiovascular disease, duration of diabetes, smoking status and corresponding baseline outcome (costs over 12 months before baseline, PAID score at baseline and EQ-5D-3L utility value at baseline, respectively) as fixed effect terms. Center effects were included as random-effects in the analysis. No time-dependent terms, interaction terms or effect modifiers were used. The difference in mean 12-month costs and outcomes were estimated based on the model. Incremental cost-effectiveness ratios (ICER) were calculated by dividing the difference in mean cost by the difference in mean health outcome. The resulting incremental cost per QALY gained was then compared against the recommended willingness-to-pay (WTP) threshold of £20,000-£30,000 per QALY by NICE [15].

A non-parametric bootstrap technique was employed to explore the uncertainty of point estimates of the difference in mean 12-month costs and outcomes from primary analyses. Five thousand bootstrapped datasets were created and the total costs and outcome estimated for each one. The results from bootstrap resampling were used to construct 95% CI for incremental costs, incremental PAID score, incremental QALYs, and to plot the cost-effectiveness plane and cost-effectiveness acceptability curve to show the uncertainty surrounding the primary results [30].

#### Sensitivity Analyses

Complete case analysis was undertaken to assess the performance of the imputation model compared to a complete case analysis that assumes data were missing completely at random.

The intervention cost per user estimated in the primary analysis was based on the number of trial participants, which would be unrealistic if the intervention were to be implemented more widely. We, therefore, undertook a one-way sensitivity analysis, exploring the cost per user as numbers of users increased.

The cost of nurse-led facilitation is per participant, so will be incurred by each registered user, regardless of the total number. Each practice referring patients to the intervention needed at least one nurse who is trained in the facilitating activities. In this trial, each practice or research nurse only undertook to facilitate activities for up to 10 patients, whereas practices are likely to have several hundred patients with T2DM who could be registered, which would reduce the per-user cost of training nurses to register patients. However, taking into consideration staff turnover and the possibility of multiple staff being trained, we made the conservative assumption that the costs of training practice staff would remain the same per user.

The costs of maintaining and delivering the intervention remain the same up to the total capacity of the current server (which is for 10,000 active users). This means that the average cost per user reduces up to 10,000 active users. The one-way sensitivity analysis, therefore, explored the change in intervention cost per user about the ICER on the implicit assumption that the impact on health services use, and QALYs in the trial was generalizable. Because the national WTP threshold is only expressed regarding incremental cost per QALY, the one-way sensitivity analysis was not undertaken for the incremental PAID score.

All analyses were undertaken using STATA SE 14.2 software.

## Results

### Participants in the Study

Recruitment took place between September 2013 and December 2014. Of 374 participants randomized, 185 were allocated to the intervention and 189 to the control group. The average age at randomization was 64.9 (SD 9.5) years old in the intervention group and 64.7 (SD 9.1) years old in the control group. There were four participants missing time since diagnosis of diabetes, two in each group. Among the participants with this information, the mean duration was 7.8 (SD 5.7) years in the intervention group and 8.2 (SD 6.1) years in the control group. The proportion of male participants in the intervention group (127/185, 69%) was similar to that in the control group (131/189, 69%). Further description of the participants’ characteristics can be found in the clinical effectiveness article [16].

### Missing Data

In the intervention group, there were 143/185 (77%) participants who completed the three months self-report questionnaire and 129/189 (70%) participants who completed the 12 months one. In the control group, there were 152/189 (80%) participants who completed the three months questionnaire and 135/189 (71%) participants who completed the 12 months questionnaire. The difference in completion rate was not significant (Pearson’s chi-squared test *P*=.459 at 3 months, *P*=.718 at 12 months). There were rare cases where participants completed the questionnaire with one or two items missing (see Multimedia Appendix 3). In general, the missing data on self-report were due to not responding. The percentage of missing data was low and did not exceed 30% for any variable. The number of imputations was therefore set to 30.

### Intervention Costs

#### Maintenance and Delivery

Staff costs of maintenance and delivery of the intervention were estimated to be £18,783 a year, including patient representatives’ feedback work (Table 2). The total infrastructure cost per year was estimated at £23,013, including £93 for 10 domain names, £3600 for website hosting, £19,200 for maintenance, and £120 for Security Socket Layer Certificate. The two parts gave a total operating cost of HeLP-Diabetes per year of £41,796. Allocating these costs to the 185 participants in the intervention group, the average cost per participant was £226.

**Table 2:** Staff costs of maintenance and delivery of HeLP-Diabetes.

Activities	Intensity	Payment scale or method	Unit cost (£) per hour	Cost (£) per year
Emails, newsletters, SMS^a^	1 day/2 weeks	Grade 6 staff	34	6675
Librarian review of recent development	1 hour/week	Fixed contract	30	1560
Patient representatives feedback on librarian’s review	30 minutes/person, 2 persons/2 weeks	Cash payment	18	468
Forum monitoring by patient representatives	30 minutes/person, 2 persons/2 weeks	Cash payment	18	468
Forum monitoring by staff	1 hour/2 weeks	Grade 6 staff	34	890
Clinical team website interaction	1 hour/time, 5 times/year	GP	121	605
Patient representatives review feedback from clinical team	15 minutes/person, 2 persons/time, 5 times/year	Cash payment	18	45
Content checking, revising and updating by staff	2 hours/2 weeks	Grade 6 staff	34	1780
Content checking, revising and updating by clinical team	2 hours/2 weeks	GP	121	6292
Total				18,783

^a^SMS: short message service.

#### Facilitating Activities

The costs of training the nurses to undertake the facilitation, including both the costs of the research staff providing the training and the costs of the nurses being trained, came to £3785 across all practices involved in the trial. The cost of the printed training materials was £78. Thus, the total training costs in the trial were £3863, or £21 per participant in the intervention group.

The time allocated to registering participants and introducing them to the intervention was 20 minutes per participant, hence cost one-third of a practice nurse’s hourly consultation rate (£44) [25]. The cost of the booklet given to each participant in the intervention group was £0.95 per booklet.

The total intervention cost was, therefore, £263 per participant, made up of £226 costs of maintenance and delivery; £21 for initial training of practice/research nurses; and £16 for nurse-led facilitation. We assumed zero costs for the comparator, as although we incurred costs during the trial, these would not have been incurred outside of a trial.

### Primary Analysis

The outcomes and the incremental cost and effectiveness of the primary analysis are presented in Table 3. The mean costs of health resources use in the 12 months trial period were higher in the control group than in the intervention group (for detailed health resources use, see Multimedia Appendix 3). The unadjusted difference in mean total costs was £12, with a lower value in the control group. When adjusted for baseline health resource use 12 months before the trial, the mean total costs in the intervention group were £131 (SE £169) higher than the control group. After further adjusting for baseline variables (ie, age, sex, history of cardiovascular diseases, smoking status, time since diabetes diagnosis), the difference was reduced to £111 (95% CI –156 to 362).

The mean PAID score was higher in the control group than the intervention group throughout the trial. The unadjusted difference in PAID score at 12 months was 3.1, with a lower score in the intervention group. After adjusting for baseline PAID score, the mean PAID score was 1.9 (SE 1.3) lower in the intervention group than the control group. This remained similar when further adjusting for other baseline variables (ie, age, sex, history of cardiovascular diseases, smoking status, time since diabetes diagnosis).

Mean EQ-5D-3L utility in both groups increased at three months from baseline. At 12 months, the mean utility value fell in both groups, but the fall was greater in the control group. Regarding QALYs, during the 12-month trial period, the intervention group had a mean QALY of 0.802 (SE 0.016) compared with 0.764 (SE 0.023) in the control group, giving an unadjusted difference of 0.038. After adjusting for EQ-5D-3L utility values at baseline and other baseline variables (ie, age, sex, history of cardiovascular diseases, smoking status, time since diabetes diagnosis), the incremental QALY was 0.020 (95% CI –0.001 to 0.044), comparing the intervention group to the control group.

**Table 3:** Incremental cost-effectiveness analysis based on imputed data, by randomized group.

Outcomes			Intervention (n=185)	Control (n=189)
**Costs (£)**				
	Intervention cost, mean		263	0
	Health resources use in the 12 months trial period^a^, mean (SE)		1816 (125)	2067 (144)
	Total cost during trial period, mean (SE)		2079 (125)	2067 (144)
	Incremental cost for intervention group, adjusted for health resources use cost at baseline and other baseline variables, mean (95% CI)		111 (–156 to 362)^b^	N/A^c^
**HRQoL^d^**				
	Baseline PAID^e^, mean (SE)		18.1 (1.3)	19.9 (1.4)
	Three months PAID, mean (SE)		15.7 (1.2)	17.3 (1.3)
	Twelve months PAID, mean (SE)		14.5 (1.2)	17.6 (1.4)
	Incremental PAID score at 12 months for intervention group, adjusted for baseline PAID score and other baseline variables, mean (95% CI)		–1.9 (–4.2 to 0.4)^b^	N/A
	Baseline EQ-5D-3L utility^f^, mean (SE)		0.793 (0.018)	0.766 (0.021)
	Three months EQ-5D-3L utility, mean (SE)		0.811 (0.016)	0.786 (0.024)
	Twelve months EQ-5D-3L utility, mean (SE)		0.793 (0.023)	0.736 (0.037)
	QALYs in the 12 months trial period^g^, mean (SE)		0.802 (0.016)	0.764 (0.023)
	Incremental QALYs for intervention group, adjusted for baseline EQ-5D-3L utility value and other baseline variables, mean (95% CI)		0.020 (–0.001 to 0.044)^b^	N/A
**ICER^h^**				
	Incremental cost (£) per unit improvement on PAID scale, mean (95% CI)		58 (–411 to 587)^b^	N/A
	Incremental cost (£) per QALY gained, mean (95% CI)		5550 (–21,077 to 52,356)^b^	N/A

^a^This was calculated based on the assumption that the medications were for chronic use and were prescribed monthly.

^b^Baseline variables included age, sex, history of cardiovascular diseases, smoking status, time since diabetes diagnosis.

^c^N/A: not applicable.

^d^HRQoL: health-related quality of life.

^e^PAID: Problem Areas in Diabetes.

^f^EQ-5D-3L: A descriptive system of health-related quality of life state.

^g^QALY: quality-adjusted life year.

^h^ICER: fully adjusted incremental cost-effectiveness ratio.

**Figure 1:**
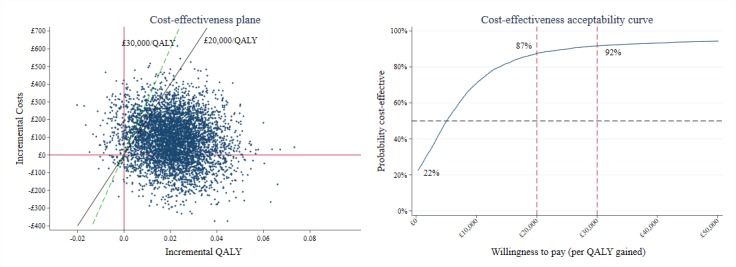
Cost-effectiveness plane and cost-effectiveness acceptability curve comparing the intervention group to the control group.

### Incremental Cost-Effectiveness Analysis

The fully adjusted incremental cost-effectiveness ratio (ICER) was £58 (95% CI –411 to 587) per unit improvement on PAID scale, and £5550 (95% CI –21,077 to 52,356) per QALY gained, comparing the intervention with the control (Table 3). For both health outcomes, the ICERs indicated a costlier and more effective intervention. Comparing with the recommended WTP threshold for QALY, the intervention presented a much lower ratio. The majority of the bootstrapped replicates fell in the north-east corner of the cost-effectiveness plane, indicating a costlier and more effective intervention (Figure 1). A lesser proportion of the replicates fell in the south-east corner, indicating a less costly but more effective intervention. A small group (153/5,000, 3%) of the replicates fell on the left side of the Y axis, indicating a less effective intervention. Overall, the majority of bootstrapped replicates fell under the WTP thresholds. The CEAC further demonstrated the conclusion was likely to be robust with an 87% probability that the intervention was cost-effective at a WTP threshold of £20,000 per QALY and 92% at £30,000 per QALY (Figure 1).

**Table 4:** Comparison of outcomes between imputed data and complete cases.

Analysis			Intervention		Control	
			n	Mean	n	Mean
**Costs of health services use (£)**						
	**In the 12 months before recruitment**					
		Imputed (SE)	185	1792 (126)	189	2084 (164)
		Complete cases (SD)	96	1793 (1,545)	101	1677 (1,418)
	**In the 12 months trial period**					
		Imputed (SE)	185	1816 (125)	189	2067 (144)
		Complete cases (SD)	96	1695 (1,404)	101	1721 (1,539)
**PAID^a^ scores**						
	**Baseline**					
		Imputed (SE)	185	18.1 (1.3)	189	19.9 (1.4)
		Complete cases (SD)	96	18.8 (16.8)	101	19.0 (16.5)
	**Twelve months**					
		Imputed (SE)	185	14.5 (1.2)	189	17.6 (1.4)
		Complete cases (SD)	96	14.6 (15.5)	101	15.9 (15.2)
**EQ-5D-3L^b^ utility**						
	**Baseline**					
		Imputed (SE)	185	0.793 (0.018)	189	0.766 (0.021)
		Complete cases (SD)	96	0.792 (0.232)	101	0.829 (0.207)
	**Three months**					
		Imputed (SE)	185	0.811 (0.016)	189	0.786 (0.024)
		Complete cases (SD)	96	0.824 (0.186)	101	0.840 (0.229)
	**Twelve months**					
		Imputed (SE)	185	0.793 (0.023)	189	0.736 (0.037)
		Complete cases (SD)	96	0.814 (0.218)	101	0.825 (0.250)

^a^PAID: Problem Areas in Diabetes.

^b^EQ-5D-3L: A descriptive system of health-related quality of life state.

**Figure 2:**
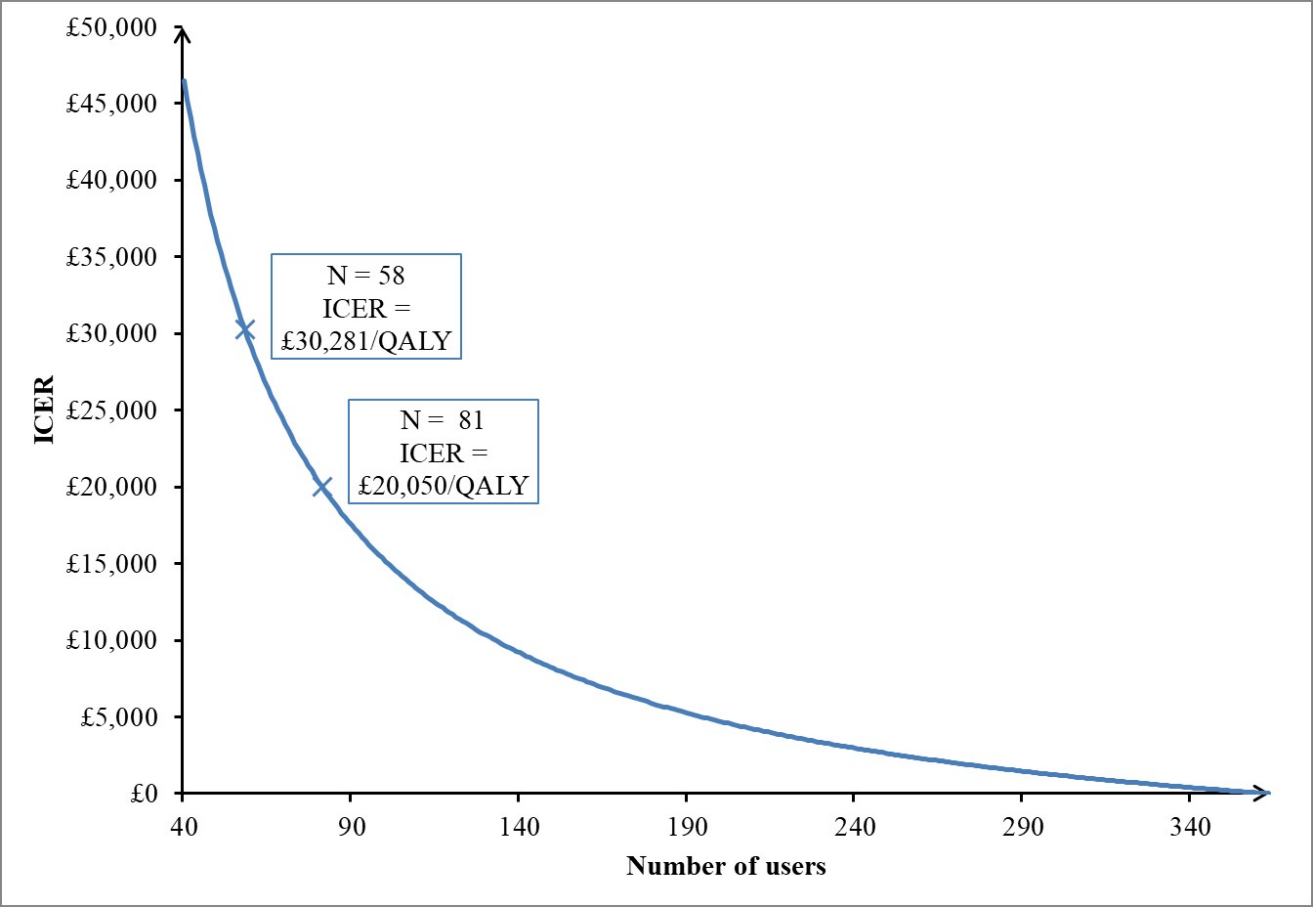
One-way sensitivity analysis of ICER in relation to number of users.

### Complete Case Analysis

A complete case analysis (CCA) was performed on 96/185 (52%) in the intervention group and 101/189 (53%) in the control group who had complete data on baseline variables required for adjustment, costs and the two health outcomes. Comparing the results from CCA to the primary analysis, the mean costs were lower in the CCA than in the primary analysis, except for the mean costs in the 12 months before recruitment in the intervention group (Table 4). Similarly, EQ-5D-3L utility scores at all time points were higher in the CCA than in the primary analysis, except for the score at baseline in the intervention group. The results of the CCA showed a smaller incremental outcomes (PAID –1.6, 95% CI –5.1 to 1.4 and QALY 0.010, 95% CI –0.018 to 0.044) and bigger incremental costs (£185, 95% CI –145 to 504), with higher variation surrounding each estimate, in comparison with the primary analysis. The ICER for a one unit improvement on PAID score was £116 (95% CI –1299 to 1690). The intervention was still cost-effective (ICER=£18,500/QALY, 95% CI -203,949 to 190,267), compared with the WTP threshold, but with a higher level of uncertainty (probability of being cost-effective was 45% at £20,000 and 52% at £30,000).

### One-Way Sensitivity Analysis for the Cost of Intervention

Assuming the effectiveness and the impact on health resources use remained the same, the ICER declined rapidly with increasing numbers of users (Figure 2). Once there were 81 users, the ICER dropped to around £20,000/QALY. The ICER became negative upon the intervention reaching 363 users, thereafter dominating the control (ie, the intervention became less costly and more effective than the control).

Figure 2. One-way sensitivity analysis of ICER in relation to the number of users

## Discussion

### Principal Results

In this within-trial economic evaluation, we found that HeLP-Diabetes plus usual care was highly likely to be cost-effective, compared with free information-only websites, such as that provided by NHS Choices or Diabetes UK, plus usual care. The ICER in the primary analysis was estimated at £5550 per QALY gained with 87% and 92% probability of being cost-effective for WTP thresholds of £20,000 and £30,000 per QALY, respectively. Once there were over 363 users, HeLP-Diabetes became dominant (ie, less costly and more effective) compared to free information websites, on top of usual care.

### Strengths and Limitations

The strength of this study comes from the reliance on actual data from a trial. We collected a wide range of information on health and social service resource use, including community, primary and secondary care. Most of the service use data came from the GP electronic health care records, supplemented by self-report data from participants for services not recorded in the electronic notes. This approach reduced the potential bias coming from retrospective recall by participants. The completeness of self-report sections was mostly secured by the mandatory questionnaire procedure which prevented skipping individual questions, therefore reduced missing data. Due to the volume of medication taken in this population, however, only the current medication at the time of data collection was extracted, and the assumption was made that all medications were for chronic use. This could overestimate medication costs, but it should not introduce a bias towards either group. The comparison between CCA and the primary analysis showed that the imputation had a greater impact on the control group than on the intervention group. The results suggested that the participants who completed all tests and questionnaires were likely to have a lower level of health services use and better health.

We decided at the outset not to include the investment costs incurred in the development of the intervention in our analysis. This was because, being funded by research grants, these costs were unlikely to re-occur if the intervention was widely implemented by the NHS, therefore being irrelevant to the decision maker for future planning. We used the running costs occurred in the trial to estimate the operating costs in practice. However, there may be more maintenance costs over a longer period as technology changes, and there may be a need for software updates. One example would be that adapting this program to optimize user experience on a mobile phone screen would incur additional programming costs. Neither PAID nor QALYs are clinical outcomes, and their association with outcomes such as glycated hemoglobin (HbA_1c_) is not always straightforward. Although necessary for comparability with other interventions, their clinical relevance is limited.

According to National Diabetes Audit 2016-17 [31], 56% of registered T2DM patients are male, and 47% are under age 65 in England. While the mean age is similar, the proportion of male patients in our study participants is higher. What impact this might have on the overall effects of HeLP-Diabetes is unknown at this point, but it is worth noting that many face-to-face self-management interventions appear to appeal more to women than men, so the gender imbalance in favour of men seen in our trial may suggest that providing both face-to-face and online self-management interventions may be one way to reach both men and women [32]. Furthermore, the participants in our study were well-managed in their condition from the beginning, with mean HbA_1c_ of 7.3% (56 mmol/mol) at baseline. In a related point, the participants in our study had a duration of diagnosis ranging from 0-34 years. However, the effectiveness data from the trial showed that neither duration of diabetes nor baseline HbA_1c_ impacted on the overall change in HbA_1c_ [16].

Population impact of an intervention is a product of effect size, reach and uptake–a highly efficacious intervention that is only used by a very small proportion of the population may have less impact than a less efficacious intervention which is widely used. These trial data cannot give us an estimate of the eventual reach and uptake of this intervention, as trial participants are known to differ from the total population of patients who may be targeted by an intervention [33]. In parallel with the trial reported here, we undertook an implementation study to explore issues around reach and uptake, along with factors which impacted on these. These data will be reported separately.

A potential disadvantage of a rigorous evaluation is the time and resource required. There may be questions about the value of such investment in a rapidly evolving landscape where the intervention in question may be rendered obsolete by the time the evaluation is completed. However, while the digital technology changes rapidly, the underlying principles do not. Another potential limitation to consider is the importance of taking into account the target population of the intervention. It is possible that benefits will not scale across the population equally. For instance, in 2017, 37% of the adults aged 65+ in Great Britain read online newspapers or magazines, in contrast to over 70% of the adults aged under 55, whilst 20% of adults aged 65+ shared self-created content online, compared with over 50% in the age groups under 55 [34]. Interventions that have a wide target population will need to reflect the heterogeneity in preferences of their target populations, and the use and impact of the interventions may vary, affecting cost-effectiveness.

### Comparison with Prior Work

Although one of the major drivers for research into digital health interventions, such as HeLP-Diabetes, is their expected cost-effectiveness [12,35], there has been relatively little published evidence. A Cochrane review of digital interventions for alcohol consumption published in 2017 identified only 7/42 (16.7%) qualified studies reported economic evaluations [36]. Several systematic reviews of economic evaluations on mental health-related digital interventions conducted in recent years identified 5 studies for anxiety disorder [37], 16 articles for mental health in general [38], and 12 studies for depression [39], respectively. Other economic evaluations of digital health interventions cover a wide range of target conditions, including irritable bowel syndrome [40], substance misuse [41], weight management [42], insomnia [43,44], eating disorders [45,46] and postoperative recovery [47]. Results of these studies were favourable regarding costs, especially when wider health care or societal costs were taken into account but did not show a significant impact on quality of life during follow-up periods. Our results are therefore broadly in line with previous findings and thus contribute to the growing but still insufficient evidence pool of economic evaluation of digital interventions.

### Conclusions

As there are 3.5 million people diagnosed with T2DM in Great Britain in 2016 [48-50], and over 90% of the households have internet access in 2017 [34], HeLP-Diabetes has the potential of delivering an effective intervention on a wide scale with negligible marginal costs. Although we do not expect internet-based interventions to be suitable for everyone at the moment, with the internet further permeating our daily life and people adapting to the internet era, there is potential for digital health interventions to help alleviate the burden of chronic conditions on health care systems in the long run. Our findings supported the cost-effectiveness of the intervention once taken up by patients. Further research is needed on how digital health interventions such as HeLP-Diabetes can be delivered and maintained in a sustainable and cost-effective manner, with the focus on user experience outside of a study setting. The successful realization of this effect might lie in identifying the more susceptible user groups and engaging them at an optimal time. More empirical studies are needed to help plan the systematic incorporation of digital interventions in medical practice.

## Appendix

Multimedia Appendix 1 Patient information sheet used in the trial.

Multimedia Appendix 2 Screenshot of HeLP-Diabetes Forum and help page.

Multimedia Appendix 3 The summary of health resources use by group, at baseline, three months and twelve months follow-ups.

Multimedia Appendix 4 CONSORT-EHEALTH checklist (V 1.6.1).

## Abbreviations

CCA: complete case analysis
EQ-5D-3L: a standardized instrument developed by the EuroQoL Group that consisted of 5 domains with 3 levels each
GP: General Practitioner
HeLP-Diabetes: healthy living for people with type 2 diabetes
ICER: incremental cost-effectiveness ratio
NHS: National Health Services
NICE: National Institute of Health and Care Excellence
NIHR: National Institute of Health Research
PAID: problem areas in diabetes
PSS: Personal Social Services
PSSRU: Personal and Social Services Research Unit
QALY: quality-adjusted life year
SMS: short message service
T2DM: type 2 diabetes mellitus
UK: United Kingdom
WTP: willingness-to-pay
