# Supplementary material for: Cost-Effectiveness of Facilitated Access to a Self-Management Website, Compared to Usual Care, for Patients With Type 2 Diabetes (HeLP-Diabetes): Randomized Controlled Trial
Source: J Med Internet Res. 2018 Jun 8;20(6):e201. doi: 10.2196/jmir.9256 (PMC6015272; doi:10.2196/jmir.9256)
Supplement: Multimedia Appendix 1 [file jmir_v20i6e201_app1.pdf]

ROYAL FREE AND UNIVERSITY COLLEGE LONDON MEDICAL SCHOOL
UCL DEPARTMENT OF PRIMARY CARE AND POPULATION HEALTH
E-HEALTH UNIT

PARTICIPANT INFORMATION SHEET

Trial of two websites to improve self-management skills in people with type 2 diabetes

You are being invited to take part in our research study. Before you make a decision, it may help to understand why the research is being done and what it would involve for you.

What is the study about?

Research has shown that diabetes education can improve people's health and wellbeing. We have designed two websites that provide diabetes education. One is a detailed website with lots of tools to help you manage your diabetes. The other is a simple website, giving the essential information everybody with diabetes needs to know. This study will test which website is better at improving glucose control and wellbeing, when used alongside usual care from your diabetes team.

Why have I been invited?

You have been invited to take part because this study is for people aged 18 or over with type 2 diabetes that are registered at your general practice.

Do I have to take part?

No. It is up to you whether to take part in the study. If you do decide to, you can let us know by: **emailing us** on this address: c.dack@ucl.ac.uk; **calling us** on this number: 020 7794 0500 (Ext: 36323); or **returning the letter of interest** in the enclosed FREEPOST envelope.

If you do not want to take part you do not need to do anything. Whether or not you choose to take part will not affect your healthcare in any way.

What will happen to me if I take part and what do I have to do?

- A nurse will contact you to check that you still want to take part and to answer any questions you might have about the study.
- If you are still happy to take part, the nurse will arrange an appointment with you. At this appointment they will take some measurements like your height, weight and blood pressure and look up some information from your medical record such as the number of appointments you have had, the number and type of medicines you are taking, and any additional problems you are experiencing as a result of your diabetes. The nurse will also take a blood sample or refer you for a blood test at the local hospital. Then you will be emailed some questionnaires to complete at home. The results of the blood tests will go into your medical record, the results of the questionnaire will not.

- After this, you will be allocated by chance (randomly) to be in one of two groups: group one will be able to use the detailed website; group two will be able to use the simple website. The nurse will arrange a second appointment to show you how to use the website you have been allocated to.
- You are then free to use the website as much or as little as you like over 12 months. To improve your self-management skills you will need to spend 15-30 minutes looking through the website each week. If you need any help using it, you can ask for this from a member of the research team.
- After 3 months, both groups will be emailed another invitation to complete some questionnaires online at home. Once you have filled these in a nurse will contact you to arrange a third appointment where they will take the same measures as before. This will happen again after 12 months.

What are the possible benefits of taking part?
Whichever group you are in, you will have the opportunity to use a website designed to improve self-management skills for 12 months. Research evidence suggests that people who are given diabetes education have better health outcomes than people who don’t. This website is only currently available to people who take part in the study.

What are the possible disadvantages of taking part?
The main disadvantage of taking part is the time it takes to meet with the nurse and complete the questionnaires. It is very unlikely that you will come to harm as a result of taking part in the study. The information on both websites has been developed by an expert team of NHS doctors, nurses, dieticians and researchers. However, some people may be upset by reading information about their health and potential future problems. If so, we encourage you to talk about any worries or anxieties you have with your doctor or nurse.

What will happen if I don’t want to carry on with the study?
You are free to change your mind about taking part at any time without giving a reason. However, any information that you have already provided will be kept in the study.

Will my taking part in this study be kept confidential?
Yes. All information collected about you during the course of the study will be kept strictly confidential. We will keep your personal identification data (your name, address) separate from the rest of the information about you in the study, which will only be identified by a unique participant identification number. We will handle, process, store and destroy data following procedures in keeping with the Data Protection Act 1998.

The results of the clinical measurements completed for the research (blood pressure, weight, blood test) will be shared with your usual health care team so you won’t need to have them done again. The results of the questionnaires you fill in will not be shared with your health care team, so if the questionnaires make you aware of a problem you should tell your GP or nurse.

Will my GP and health care team know I am in the study?
Yes. We will inform your GP that you are in the trial. This will not affect the care they give you in any way.

What personal data do I have to provide and what will it be used for?
Some information about your use of the HeLP-Diabetes website will be automatically recorded (e.g. which sessions you use). This will help us to see how people use the website. You will also be asked to give some basic information about yourself (e.g. gender, email address) which will be used to create your personal account, and give you personalised advice. Other information is asked for because it will help the researchers to understand if the website is useful and who it works best for. Your contact details will only be used to contact you while the study is running and to send you a summary of the research findings (if you want one). Nobody outside the research team will see information that you enter into the website.

What will happen to the blood samples I give as part of the study?
The blood samples taken from you as part of the study will be analysed at the local hospital where the rest of your blood tests are analysed. They will be disposed of in accordance with the Human Tissue Authority's Code of Practice after the results have been sent to your GP.

What happens when the study ends?
We hope that the results of the study will influence NHS policy so that everyone with diabetes in England can have access to the better of the two websites.

What will happen to the results of the research study?
At the end of the study we will write a report of our results to be published in medical journals and presented at conferences. We would be happy to send you a summary of the results if you wish to see this.

Who is organising and funding the research?
This study is organised by researchers from the Departments of Primary Care and Population Health at University College London. The National Institute of Health Research (reference RP-PG-0609-10135) has funded this project. For more information about the research team, please see http://www.ucl.ac.uk/pcph/research-groups-themes/e-health

Who has reviewed the study?
This study has been reviewed and given favourable opinion by the Camden and Islington National Research Ethics Committee (reference 12/LO/1571). The study is sponsored by University College London.

What if there is a problem?
If you wish to complain, or have concerns about any part of this study please contact Dr Charlotte Dack (Phone: 020 7794 0500 ext 36323, email: c.dack@ucl.ac.uk) who will do her best to answer your questions. If she cannot help, your concern will be passed to the Chief

Investigator, Professor Elizabeth Murray, who has overall responsibility for the study. The contact details for both Dr Dack and Professor Murray are below. If you are still unhappy and wish to complain formally, you can do this through the NHS Complaints procedure. Details can be obtained from the Patient Advice and Liaison Service (PALS - www.pals.nhs.uk).

Is there an independent contact point where I can get general advice about taking part in research?
Yes. INVOLVE is a national advisory group that provides advice on public involvement in research. You can find out more from their website: www.invo.org.uk

You can contact them at: INVOLVE, Wessex House, Upper Market Street, Eastleigh, Hampshire, SO50 9FD or Telephone: 023 8065 1088

Further information and contact details
If you have any questions at all about the study or would like further information, please contact the trial manager, Dr Charlotte Dack using the contact details below.

Name	Dr Charlotte Dack	Professor Elizabeth Murray –
Role	Trial Manager	Chief Investigator
Tel	020 7794 0500 ext 36323	020 7794 0500 ext 36747
Email	c.dack@ucl.ac.uk	elizabeth.murray@ucl.ac.uk
Address	eHealth Unit,<br>UCL Research Department of Primary Care & Population Health<br>Upper 3rd Floor, Royal Free Hospital, Rowland Hill Street<br>London NW3 2PF	
Fax	020 7794 1224	
Web	https://www.ucl.ac.uk/pcph/research-groups-themes/e-health	

What do I do if I wish to take part?
If you are interested in taking part in the study please fill in the **letter of interest** enclosed and return it in the **stamped addressed envelope** or contact **Charlotte Dack** using the details above.

THANK YOU FOR YOUR TIME IN READING THIS INFORMATION SHEET
