# Supplementary figures and images for: Cost-Effectiveness of Facilitated Access to a Self-Management Website, Compared to Usual Care, for Patients With Type 2 Diabetes (HeLP-Diabetes): Randomized Controlled Trial
Source: J Med Internet Res. 2018 Jun 8;20(6):e201. doi: 10.2196/jmir.9256 (PMC6015272; doi:10.2196/jmir.9256)

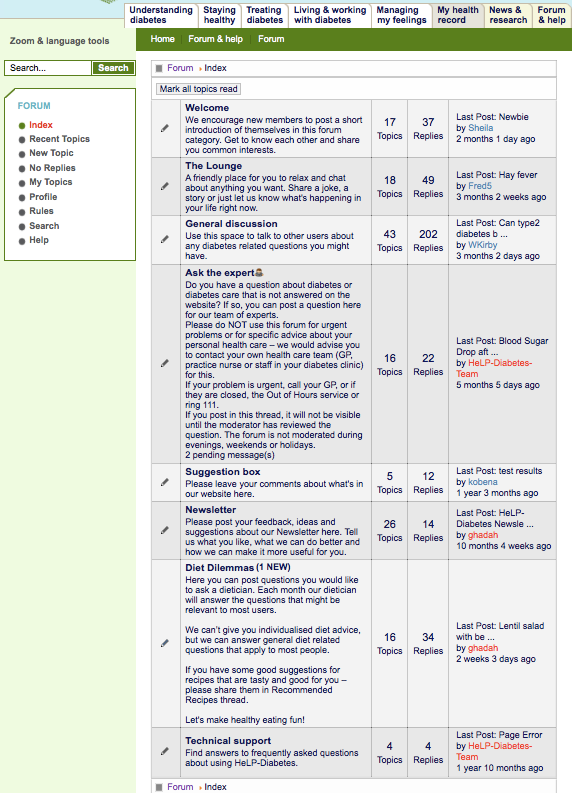

Supplement: Multimedia Appendix 2 [file jmir_v20i6e201_app2.png]
