# Supplementary material for: Cost-Effectiveness of Facilitated Access to a Self-Management Website, Compared to Usual Care, for Patients With Type 2 Diabetes (HeLP-Diabetes): Randomized Controlled Trial
Source: J Med Internet Res. 2018 Jun 8;20(6):e201. doi: 10.2196/jmir.9256 (PMC6015272; doi:10.2196/jmir.9256)
Supplement: Multimedia Appendix 3 [file jmir_v20i6e201_app3.pdf]

The health resource use data collected at baseline, three months and twelve months follow-ups, covered the time periods of 12 months before baseline, months 1-3 of the trial and months 4-12 of the trial. Limited by the lost-to-follow-up, the mean usage at the two follow-up points couldn't be combined and compared directly. However, the large standard deviations indicated that the use of health services was not even among participants. Rather, a small group of participants appeared to be use a very high volume of services. Details were presented in Supplemental Table 1.

Supplemental Table 1 Frequency of health care and social services use at each time point, by group

	**12 months prior to baseline**		**Months 1-3**		**Months 4-12**	
**Service item**	**Intervention**	**Control**	**Intervention**	**Control**	**Intervention**	**Control**
	**Mean (S.D.)**	**Mean (S.D.)**	**Mean (S.D.)**	**Mean (S.D.)**	**Mean (S.D.)**	**Mean (S.D.)**
**Services use recorded by nurses**	**n = 185**	**n = 189**	**n = 153**	**n =165**	**n = 151**	**n = 155**
GP consultation (in surgery)	4.65 (4.60)	5.22 (4.54)	1.39 (1.74)	1.71 (1.99)	2.68 (2.78)	3.10 (2.97)
GP consultation (home visit)	0.02 (0.18)	0.05 (0.47)	0	0.02 (0.13)	0.01 (0.08)	0.07 (0.52)
GP consultation (telephone)	1.01 (1.78)	1.76 (3.31)	0.42 (0.89)	0.57 (1.05)	0.91 (1.51)	1.30 (2.14)
Practice nurse consultation (in surgery)	3.57 (4.51)	3.43 (4.82)	1.38 (1.53)	1.40 (1.96)	2.50 (3.14)	2.61 (3.99)
Practice nurse consultation (home visit)	0.02 (0.23)	0.10 (1.38)	0.18 (1.95)	0.01 (0.08)	0	0.03 (0.16)
Walk-in centre	0.04 (0.28)	0.06 (0.30)	0.03 (0.26)	0.02 (0.15)	0.08 (0.67)	0.10 (0.32)
Out of Hour service (telephone)	0.03 (0.19)	0.06 (0.32)	0.02 (0.18)	0.02 (0.13)	0.03 (0.18)	0.08 (0.34)
Out of Hour service (home visit)	0.02 (0.16)	0.02 (0.18)	0	0	0	0.01 (0.16)
Out of Hour service (clinics)	0.06 (0.32)	0.09 (0.35)	0.03 (0.02)	0.02 (0.15)	0.12 (0.65)	0.08 (0.41)
Clinical test	15.59 (28.95)[a]	17.30 (32.13)	3.62 (4.66)	3.27 (3.36)	7.42 (5.14)	7.17 (4.80)[a]
A & E admission	0.15 (0.45)	0.26 (0.68)	0.07 (0.31)	0.05 (0.25)	0.10 (0.36)	0.15 (0.47)
Outpatient attendance	2.15 (2.89)	2.88 (4.16)	0.81 (1.33)	0.79 (1.21)	1.33 (2.64)	1.54 (2.10)
Day case	0.16 (0.47)	0.15 (0.46)	0.03 (0.16)	0.05 (0.22)	0.13 (0.47)	0.12 (0.38)
Inpatient admission	0.14 (0.47)	0.17 (0.72)	0.05 (0.25)	0.01 (0.11)	0.06 (0.26)	0.10 (0.33)
Podiatrist attendance	0.37 (1.19)	0.35 (1.19)	0.10 (0.39)	0.10 (0.34)	0.30 (0.96)	0.18 (0.83)[a]
Eye screening[b]	0.91 (0.43)	0.96 (0.42)	0.34 (0.48)	0.32 (0.46)	0.58 (0.51)	0.65 (0.48)
Physiotherapy	0.32 (1.29)	0.27 (1.08)	0.15 (0.62)	0.16 (0.84)	0.21 (1.06)[a]	0.36 (1.38)[a]
Counselling	0.18 (1.14)	0.08 (0.70)	0.03 (0.27)	0.02 (0.17)	0.09 (0.57)	0.01 (0.11)[a]

service						
**Services use reported by participants**	**n = 185**	**n = 189**	**n = 143**	**n = 152**	**n = 129**	**n = 135**
District nurse consultation (home visit)	0.11 (0.96)	0.19 (1.55)	0	0.01 (0.08)	0	0.01 (0.09)
District nurse consultation (clinic)	0.58 (1.16)	0.75 (2.07)	0.29 (0.61)	0.36 (0.77)	0.45 (0.85)	1.07 (4.35)
NHS Direct	0.20 (0.84)	0.28 (0.93)	0.06 (0.32)	0.10 (0.55)[c]	0.12 (0.50)	0.10 (0.39)
Counselling service	0.12 (0.73)	0.29 (1.49)	0.05 (0.32)[a]	0.08 (0.42)	0.13 (0.76)	0.19 (0.99)
Social worker	0.01 (0.07)	0.06 (0.53)	0	0	0.02 (0.18)	0
Occupational therapy	0.24 (2.06)	0.11 (0.65)	0.01 (0.08)	0.05 (0.50)	0.05 (0.33)	0.07 (0.38)[d]
Dietician attendance	0.21 (0.77)	0.41 (1.87)	0.05 (0.25)	0.14 (1.25)	0.15 (0.78)	0.12 (0.49)

[a] One person had missing data on this item

[b] The majority of the eye screening was undertaken by a community retinal screening service, with 10.5% taken in an optician and 30.4% in hospital in the intervention group and 10.4% in an optician and 28.6% in hospital in the control group.

[c] Two people had missing data on this question

[d] One person had missing data on all other questions except for this one
